# Revisiting the Small Heat Shock Protein Family in *Caenorhabditis elegans:* Insights from Phylogenetic, Structural, and Functional Analyses

**DOI:** 10.17912/micropub.biology.002271

**Published:** 2026-08-11

**Authors:** Rushali Kamath, Prasad Kasturi

**Affiliations:** 1 School of Biosciences and Bioengineering, Indian Institute of Technology Mandi, Mandi, HP, India

## Abstract

Small heat shock proteins (sHSPs) are ATP-independent molecular chaperones with diverse cellular functions. Here, we systematically reassessed two subfamilies of sHSPs in *C. elegans* by integrating evolutionary, genomic, and biophysical analyses. We report analysis of 18 α-crystallin domain-containing sHSPs, including two previously uncharacterized HSP-16-like proteins (designated *hsp-16.31* & *hsp-16.32* ). This analysis also supports the inclusion of two other proteins, ZK1128.7 and Y55F3BR.6 , as additional members of the *C. elegans* sHSP family. Comparative analyses reveal substantial diversity in tissue expression, intrinsic disorder, and liquid-liquid phase separation (LLPS) propensity, highlighting extensive functional specialization within the family.

**Figure 1. Caenorhabditis elegans small heat shock proteins:** (A) Multiple sequence alignment of all the 18 sHSPs, with only the a-crystallin domain shown with colours representing specific conserved amino acids. HSP-43 is highlighted (B) Phylogenetic tree constructed using amino acid sequencesand the percentages indicate sequence similarity, while the numbers indicate branch length. (C) Chromosomal loci of the sHSPs. (D) Tissue specific expression or localization of the sHSPs. This image was generated using BioRender. (E) Intrinsic disorder and LLPS propensities of the sHSPs. Disorder predictions by IUPred2A are shown in red and predictions by PrDOS are shown in orange. LLPS propensities predicted by catGRANULE are light blue and FuzDrop predictions are in dark blue. Table 1. Details of Caenorhabditis elegans small heat shock proteins.

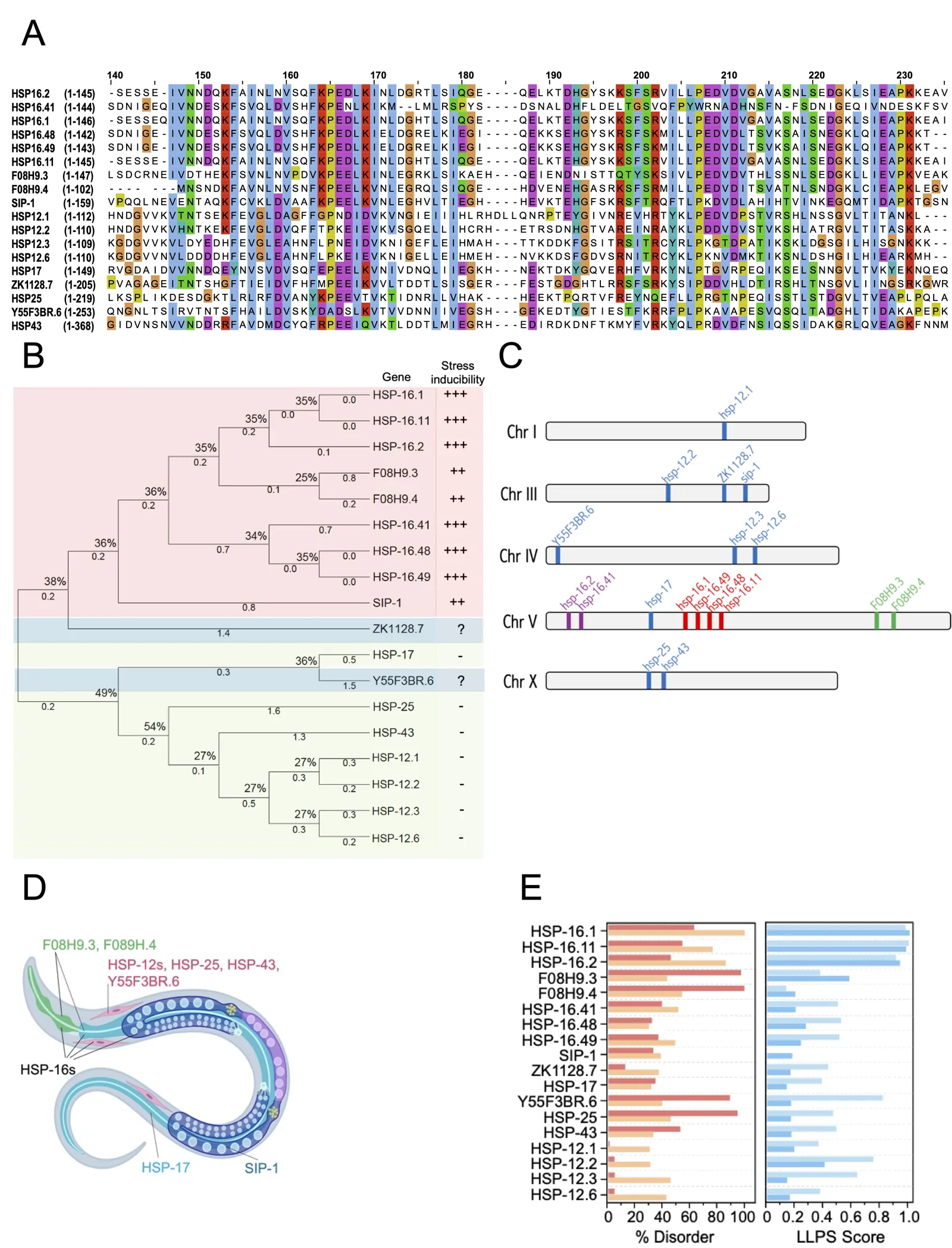



## Description

Small heat shock proteins (sHSPs) are an evolutionarily conserved family of ATP-independent molecular chaperones that play roles in maintaining protein homeostasis under both physiological and stress conditions. Members of this family are characterized by the presence of a conserved α-crystallin domain (ACD) and variable N- and C-terminal regions (Haslbeck et al., 2005; Basha et al., 2012) with strong roles in oligomerization and “holding” a vast repertoire of proteins during stress. To redefine the *C. elegans* sHSP family, we systematically identified proteins containing the conserved ACD and classified them into subfamilies using sequence conservation and phylogenetic analyses. We further report their chromosomal location, tissue-enriched expression, intrinsic disorder, and predicted liquid–liquid phase separation (LLPS) propensity to comprehensively characterize the family.

We report an analysis of 18 proteins with a predicted α-crystallin domain, although the *C. elegans* sHSP family is commonly described as containing 16 members (Iburg et al., 2020; Strauch et al., 2023). Multiple sequence alignment demonstrates that these proteins possess the characteristic ACD and conserved residues shared by other HSP-16 family members with smaller N-termini ( Figure 1A ). These proteins comprise both constitutively expressed and heat stress-inducible members and are classified into two subfamilies based on their sequence similarity and patterns of stress inducibility ( Figure 1B, Table 1).

The first subfamily comprises the six well-characterized HSP-16 proteins, which are organized into two genomic clusters and are rapidly induced in response to heat shock (Stringham et al., 1992; Burnaevskiy et al., 2019) and during aging (Walther et al., 2015). Consistent with previous reports, these canonical HSP-16 proteins form a highly conserved subgroup ( Figure 1B ). We further expand this subgroup by identifying two additional HSP-16-like proteins, F08H9.3 and F08H9.4 , which share high sequence similarity with the canonical HSP-16 proteins. These genes are located within a third genomic cluster on the same chromosome as the canonical *hsp-16* loci ( Figure 1C ) (van den Berg et al., 2025). Previous studies have shown that they exhibit weak and delayed induction following heat stress, with expression restricted to specific tissues ( Figure 1D ; Table 1) (Shim et al., 2003). Phylogenetic analysis places both proteins within the HSP-16 clade, suggesting that they originated through gene duplication events within the HSP-16 family (Aevermann and Waters, 2008). According to the Alliance of Genome Resources, these genes are currently designated *hsp-16.2 0* and *hsp-16.2 1* . To avoid potential confusion with the established gene *hsp-16.2* , we propose renaming them *hsp-16.31* and *hsp-16.32* , respectively. This nomenclature reflects their genomic organization and evolutionary relationship within the HSP-16 subfamily while distinguishing them as members of a separate genomic cluster from the canonical *hsp-16* genes. In addition to the canonical HSP-16 proteins, the non-canonical sHSP, SIP-1 , also belongs to this subfamily and exhibits stress-inducible expression. Predominantly expressed in the germline and oocytes, SIP-1 is a pH-sensitive sHSP that remains inactive under normal physiological conditions but is activated by intracellular acidification (Fleckenstein et al., 2015).

The second subfamily comprises the HSP-12 proteins, HSP-17 , HSP-25 , HSP-43 , and two other uncharacterized proteins (ZK1128.7 and Y55F3BR.6 ) ( Figure 1B ). Unlike the HSP-16 subfamily, these non-canonical sHSPs are generally not induced by heat stress (Fu et al., 2021) and likely represent functionally specialized members that have diverged from the canonical stress-responsive sHSPs. This HSP-12 subfamily is both structurally and functionally distinct from the HSP-16 subfamily. Among its members, HSP-12.6 is a notable structural outlier (Leroux et al., 1997). Although it contains the conserved ACD, it possesses the shortest N- and C-terminal regions among known sHSPs, does not form large oligomeric assemblies, and lacks general chaperone activity against aggregation-prone proteins in vitro. Instead, HSP-12.6 exhibits highly selective protective activity by binding myosin-containing thick filaments while excluding thin filaments and exogenous aggregation-prone proteins (Fern et al., 2026).

Among the non-canonical *C. elegans* sHSPs, HSP-17 and HSP-25 have evolved distinct specialized functions. HSP-17 functions as a selective "aggregase," forming large supramolecular assemblies at elevated temperatures and acting as a constitutively active chaperone that can either suppress or promote protein aggregation in a substrate-dependent manner (Zhang et al., 2015; Iburg et al., 2020; Strauch et al., 2023). In contrast, HSP-25 serves primarily as a structural protein, localizing to dense bodies and M-lines of the body-wall muscle. Remarkably, an N-terminal deletion variant retains both oligomerization and chaperone activity, demonstrating that the ACD alone is sufficient to support its core sHSP functions (Ding & Candido, 2000; Guo & Cooper, 2000).

Although HSP-43 is more divergent and has received comparatively little attention as an sHSP, it contains the conserved ACD and clusters with other *C. elegans* sHSPs in phylogenetic analyses. Together, these structural and evolutionary features support its classification as a bona fide small heat shock protein. Consistent with this classification, HSP-43 has been reported to interact with HSP-25 and the previously uncharacterized sHSP Y55F3BR.6 , suggesting a functional association among these proteins.

ZK1128.7 represents perhaps the most intriguing member of the family. Based on sequence similarity and phylogenetic analyses, it clusters within the HSP-16 subfamily ( Figure 1B ). However, STRING network analysis predicts functional associations primarily with members of the HSP-12 subfamily. This apparent discrepancy suggests that ZK1128.7 may represent an evolutionary intermediate, retaining sequence characteristics of the HSP-16 subfamily while sharing functional interactions with the non-canonical HSP-12 subfamily. Such a position may provide insights into the evolutionary diversification and functional specialization of *C. elegans* sHSPs.

The remarkable client diversity of sHSPs is thought to arise, at least in part, from their intrinsically disordered regions and their ability to undergo LLPS, both of which facilitate dynamic protein–protein interactions (Crotti et al., 2026). To gain further insight into the biophysical properties of the *C. elegans* sHSP family, we compared the predicted intrinsic disorder content and LLPS propensity of all 18 members ( Figure 1E ; Table 1). The sHSP family exhibited considerable heterogeneity in both predicted intrinsic disorder and LLPS propensity. Several proteins, including HSP-16.1 , HSP-16.1 1, HSP-16.2 , HSP-25 , and Y55F3BR.6 , exhibited relatively high levels of both intrinsic disorder and LLPS propensity. In contrast, *hsp-16.2 0* ( F08H9.3 ) and *hsp-16.2 1* ( F08H9.4 ) were predicted to be almost entirely intrinsically disordered yet exhibited only modest LLPS propensity, indicating that intrinsic disorder alone is not sufficient to predict phase-separation behavior. Conversely, HSP-17 and most members of the HSP-12 subfamily displayed low-to-moderate intrinsic disorder and LLPS propensity, highlighting the diverse biophysical strategies that have evolved within the *C. elegans* sHSP family. Among these, Y55F3BR.6 was identified as an interacting partner of HSP-43 and HSP-25 in muscle tissue (Fu et al., 2020), and its high predicted intrinsic disorder and LLPS propensity suggest a potential role in the sequestration or organization of protein assemblies. Its phylogenetic relationship to HSP-17 , together with its interaction with the similarly disordered HSP-25 , further supports the hypothesis that Y55F3BR.6 may participate in dynamic client interactions and contribute to the formation of higher-order protein assemblies.

Collectively, these analyses provide an updated framework for understanding the *C. elegans* sHSP family by integrating evolutionary, genomic, structural, and biophysical characteristics. We establish a revised classification comprising two major subfamilies, the HSP-16 and HSP-12 subfamilies; expand the HSP-16 subfamily by identifying two previously unrecognized HSP-16-like proteins for which we propose the nomenclature *hsp-16.31* and *hsp-16.32* ; and support the classification of HSP-43 as a bona fide sHSP based on its conserved ACD and evolutionary relationship with other family members. We also highlight the previously uncharacterized proteins ZK1128.7 and Y55F3BR.6 as promising candidates for understanding the evolutionary diversification and functional specialization of the sHSP family. Furthermore, our analyses reveal substantial diversity among the 18 *C. elegans* sHSPs in tissue-enriched expression, intrinsic disorder, and LLPS propensity, underscoring the remarkable structural and functional heterogeneity of this protein family. Together, our study provides a comprehensive and updated framework for the *C. elegans* sHSP family by integrating revised nomenclature, phylogenetic classification, and structural and biophysical characteristics that are likely to underlie the functional diversity of individual sHSPs.

## Methods

*Sequence Alignment and Phylogenetic Analysis*

Verified sHSP sequences were retrieved from the National Center for Biotechnology Information database and WormBase. Multiple sequence alignment was performed using the Clustal Omega algorithm and visualized in Jalview 2.11.5.1 (Waterhouse et al., 2009). Conserved regions, including the characteristic ACD, were identified and visualized based on the alignment consensus. The aligned sequences were subsequently analyzed using MEGA11 (Tamura et al., 2021), and a phylogenetic tree was constructed using the Maximum Likelihood method with default parameters. Evolutionary distances and sequence similarities were used to infer the relationships among sHSP family members, and branch lengths were mapped according to sequence divergence.

*Assessment of Stress-Responsive Induction*

Stress inducibility of the sHSP genes was assessed through a survey of the published literature. For each sHSP, we searched for studies reporting transcriptional induction in response to heat stress. A gene was classified as heat stress-inducible if the original study reported a statistically significant increase in its expression under heat stress compared with control conditions.

*Graphical Representation of Tissue Localization of sHSPs*

Tissue localization data for sHSPs were compiled through literature search and by querying the Tissue Enrichment Analysis (TEA) tool available in WormBase (https://wormbase.org/tools/enrichment/tea/tea.cgi). Tissue-specific expression and enrichment information were integrated and used to generate graphical representations illustrating the tissue localization patterns of the various sHSP family members (Angeles-Albores et al., 2016).

*Intrinsic Disorder and LLPS Propensity Prediction*

The intrinsic disorder propensity of sHSP proteins was predicted using IUPred 2A (Mészáros et al., 2018) and PrDOS server (Ishida and Kinoshita, 2007). Residue-wise disorder scores were obtained, and the percentage of intrinsically disordered residues was calculated for each protein. Liquid–liquid phase separation (LLPS) propensity was assessed using the catGRANULE 2.0 ROBOT (https://tools.tartaglialab.com/catgranule2) 2.0 platform (Monti et al., 2025) and FuzDrop (Vendruscolo et al., 2026). The predicted disorder content and LLPS propensity scores were subsequently visualized and analyzed using OriginPro 2024.

Table-1: *Details of Caenorhabditis elegans small heat shock proteins.*

**Table:** 

**SNo**	**Gene name**	**Molecular** **Weight (Da)**	**WormBase ID**	**Uniprot ID**	**Chromosomal Loci**	**Tissue enrichment/ Sub-cellular Localization**	**Heat stress induction**	**Disorder % IUPred**	**Disorder % PrDOS**	**LLPS Score- CAT-granule**	**LLPS Score- FuzDrop**
1	*hsp-12.1*	12528.06	WBGene00011906	A0MSV7; O01263; G5EE65	I:10581538...10582615	Striated muscle dense body	No	0.892	17.857	0.67	0.1494
2	*hsp-12.2*	12264.82	WBGene00002011	P34328	III:8138077…8139631	Body wall muscle	No	4.545	19.09	0.84	0.1336
3	*hsp-12.3*	12293.92	WBGene00002012	Q20164	IV:9444791…9445314	Muscle	No	4.587	12.844	0.79	0.3816
4	*hsp-12.6*	12620.43	WBGene00002013	Q7JNB0; G5EE36	IV:9446229…9447228	Uterine seam cell	No	4.545	12.727	0.675	0.1802
5	*hsp-16.2*	16242.4	WBGene00002016	P06582: V6CLQ2	V:1804334…1804957	Intestine and pharynx	Yes	45.517	13.793	0.91	0.1605
6	*hsp-16.1*	16381.42	WBGene00002015	P34696	V:9087293…9087782	Intestine, nervous system	Yes	60.959	19.178	0.94	0.1577
7	*hsp-16.11*	16253.29	WBGene00002017	P34696	V:9090077… 9090604	Intestine, nervous system	Yes	53.793	16.551	0.95	0.1586
8	*hsp-16.41*	16380.45	WBGene00002018	P06581	V:1805178…1805875	Intestine and Pharyngeal tissue	Yes	38.888	13.194	0.73	0.1298
9	*hsp-16.48*	16162.19	WBGene00002019	P02513	V:9088131...	Muscle and Hypodermis	Yes	31.69	15.492	0.74	0.1553
10	*hsp-16.49*	16299.33	WBGene00002020	P02513	V:9089280…	Muscle and Hypodermis	Yes	36.363	16.083	0.735	0.1659
11	*F08H9.3* * ( hsp-16.2 0) *	16242.4	WBGene00008591	Q19227	V:14461947…14463338	Pharynx	Yes, weakly	96.598	20.547	0.675	0.2245
12	*F08H9.4* * ( hsp-16.2 1) *	11296.66	WBGene00008592	Q19228	V:14463520…14464428	Excretory canal and a few neuronal cells	Yes	99.019	12.5	0.57	0.2552
13	*hsp-17*	17573.71	WBGene00002021	Q7JP52; Q20660	V:8384799… 8385929	Intestine and excretory canal	Physiological expression	34.228	21.476	0.68	0.1889
14	*sip-1*	17839.21	WBGene00004798	Q20363	III:10506106…10506801	Oocytes and Embryos	Yes	32.704	22.641	0.51	0.1861
15	*ZK1128.7*	22988.32	WBGene00014233	G5EF99	III:10137006..10139124	Unknown	Unknown	12.195	18.048	0.7	0.5431
16	*hsp-25*	25256.04	WBGene00002023	Q17849; Q5H9M9: Q86GU1	X:6034463… 6039644	Dense Bodies and M-lines of Body Wall Muscle: Pharynx and Spermatheca	Physiological expression	94.064	36.073	0.715	0.8808
17	*Y55F3BR.6*	27401.44	WBGene00021943	Q9N350; A0A1I6CMC9	IV:835033… 842126	M-lines and dense bodies of the body wall muscles	Unknown	88.537	32.015	0.87	0.9219
18	*hsp-43*	43241.55	WBGene00002024	B0M0L8; H2KYS1	X:6233151… 6235543	M-lines and dense bodies of the body wall muscles	Yes	52.445	41.847	0.725	0.9447
